# A Papillary Thyroid Microcarcinoma Revealed by a Single Bone Lesion with No Poor Prognostic Factors

**DOI:** 10.1155/2013/719304

**Published:** 2013-02-20

**Authors:** Yann Godbert, Benedicte Henriques-Figueiredo, Anne-Laure Cazeau, Xavier Carrat, Marc Stegen, Isabelle Soubeyran, Francoise Bonichon

**Affiliations:** ^1^Department of Nuclear Medicine, Institut Bergonié, 229 Cours de L'Argonne, 33076 Bordeaux, France; ^2^Department of Radiotherapy, Institut Bergonié, 229 Cours de L'Argonne, 33076 Bordeaux, France; ^3^Department of Surgery, Institut Bergonié, 229 Cours de L'Argonne, 33076 Bordeaux, France; ^4^Department of Nuclear Medicine, Centre Hospitalier de la Côte Basque, 13 avenue de l'interne Jacques Loëb, 64109 Bayonne, France; ^5^Department of Pathology, Institut Bergonié, 229 Cours de L'Argonne, 33076 Bordeaux, France

## Abstract

*Objectives*. Thyroid carcinomas incidence, in particular papillary variants, is increasing. These cancers are generally considered to have excellent prognosis, and papillary microcarcinomas are usually noninvasive. Many prognostic histopathology factors have been described to guide therapeutic decisions. Most patients are treated with total thyroidectomy without radioiodine treatment or partial surgery. *Case Summary*. A 65-year-old man with no significant medical history presented with pain in the left chest wall that had been present for several months. A computed tomography (CT) found a large tissue mass of 4 cm responsible for lysis of the middle arch of the 4th rib on the left. It was a single lesion, highly hypermetabolic on the 18-FDG PET/CT. The histology analysis of the biopsy and surgical specimen favored an adenocarcinoma with immunostaining positive for TTF1 and thyroglobulin (Tg). The total thyroidectomy carried out subsequently revealed a 4 mm papillary microcarcinoma with vesicular architecture of the right lobe, well delimited and distant from the capsule without vascular embolisms. After two radioiodine treatments, the patient is in complete clinical, biological, and radiological remission. *Conclusion*. This extremely rare case of a singular bone metastasis revealing a papillary thyroid microcarcinoma illustrates the necessity of further research to better characterize the forms of papillary thyroid microcarcinomas with potentially poor prognosis.

## 1. Introduction 

Papillary thyroid microcarcinomas (PTMCs) are defined according to the World Health Organisation (WHO) as small papillary thyroid carcinomas (PTCs) less than 10 mm in size [1]. Due to the increasing use of cervical ultrasound and fine needle aspiration, the incidence of PTMCs is constantly rising, and prognosis is considered to be excellent [2]. Various prognostic factors have been described, mostly linked to the presence or absence of cervical lymph node involvement. Metastatic risk in PTMCs is extremely low [3] and generally only affects pulmonary parenchyma. 

Here, we describe a case of PTMC with no poor prognostic histological criteria that was discovered through a single secondary costal lesion. 

## 2. Case Presentation

**Figure 1:**
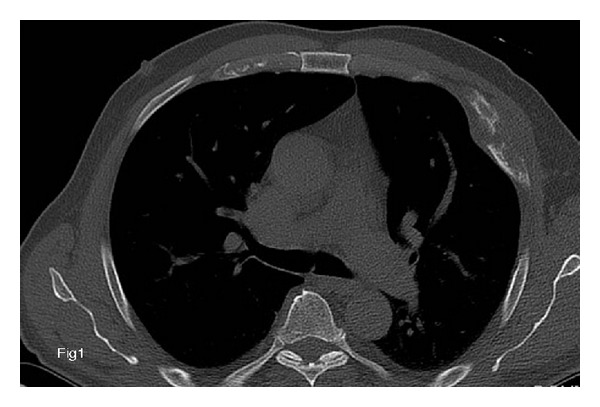
Axial CT scan for 65-year-old man with papillary thyroid microcarcinoma: large lytic mass of the anterior arch of the left 4th rib with infiltration of the soft tissues.

**Figure 2:**
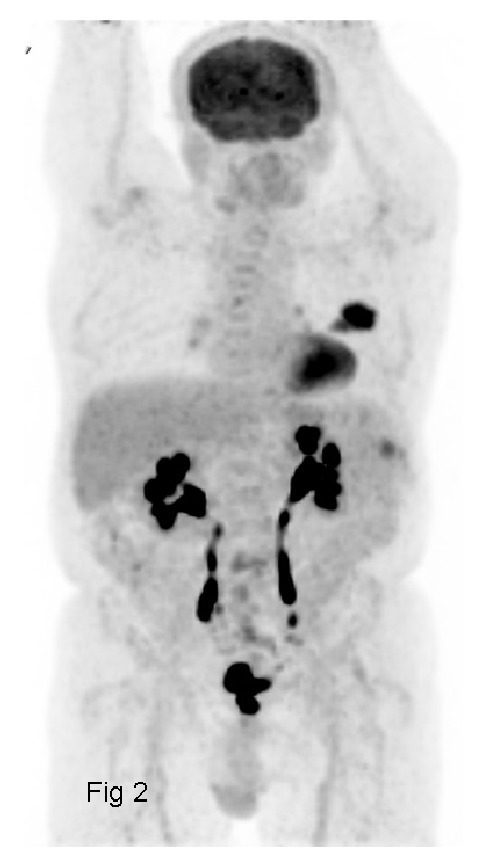
Whole-body 18FDG PET/CT image for 65-year-old man with papillary thyroid microcarcinoma reveals single hypermetabolic lytic mass in the fourth rib.

The patient was a 65-year-old man with no significant medical background, who presented with pain in the left chest wall that had been present for several months (April 2011). X-rays and the computed tomography (CT) scan revealed a large lytic mass on the middle arch of the left 4th rib (Figure 1). This hypermetabolic osteolysis was the only tumor identified on the 18-Fluoro-Deoxy-Glucose-(FDG-) positron emission tomography (PET)/CT scan (Figure 2). In June 2011, a biopsy then a surgery involving parietectomy was carried out. The anatomopathology analyses revealed a papillary adenocarcinoma with immunostaining positive for TTF1 and thyroglobulin. The metastasectomy was wide with a multicostal resection and clear margins (Figure 4). No adjuvant radiotherapy was performed. 

**Figure 3:**
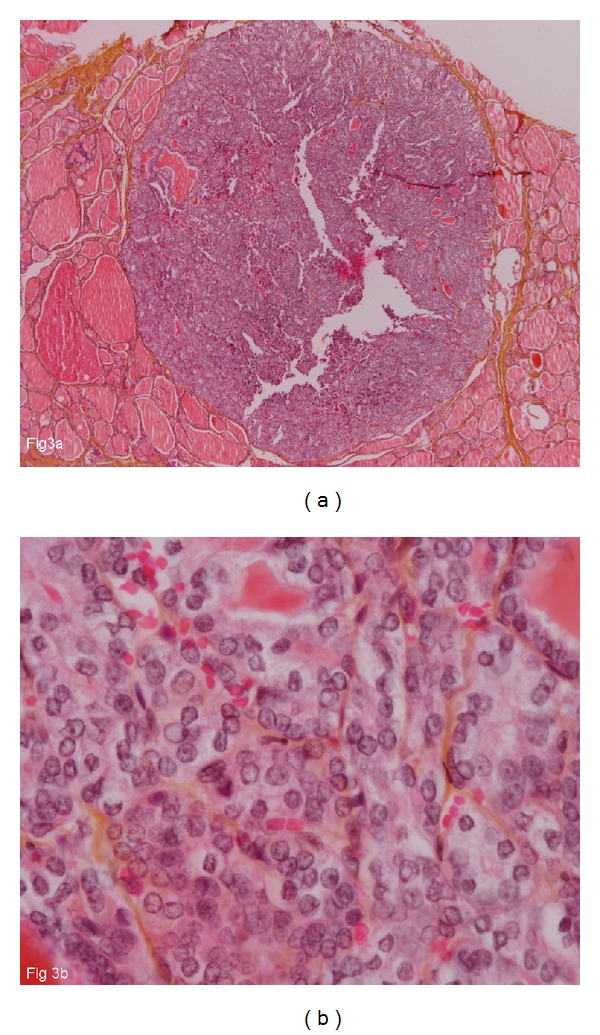
65-year-old man with papillary thyroid microcarcinoma. The tumor has a follicular architecture, measures 4 mm in diameter, and is well circumscribed (HES, Gx25). Inset shows characteristic nuclear features with nuclear overlapping, clearing, and irregular contours (HES, Gx400).

**Figure 4:**
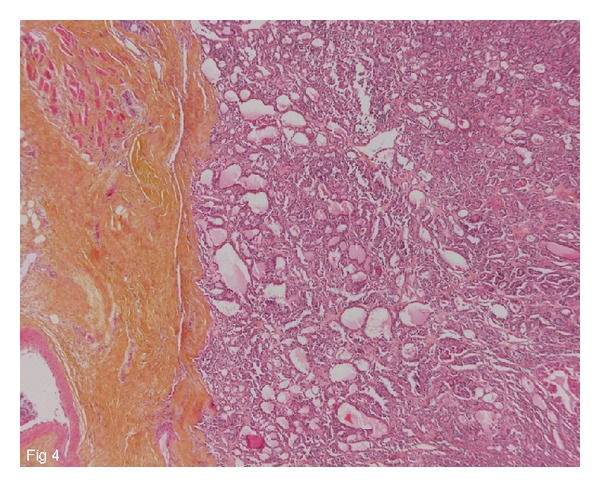
65-year-old man with papillary thyroid microcarcinoma metastatic to chest wall. The tumor infiltrates soft tissue (HES, Gx25) with clear margins.

After discussion in a multidisciplinary meeting, it was decided to perform a total thyroidectomy with central and laterocervical lymph node clearance on the right side (8 mm nodule at the right lobe of the thyroid). The surgery was performed in July 2011 with no postoperative complications. The histological examination of the surgical specimen revealed a PTMC with follicular architecture of 4 mm in the right lobe, encapsulated, well delimited, and distant from the thyroid capsule, with no vascular invasion (Figure 3). There was no lymphoid involvement (stage pT1a pN0 M1). Molecular testing was done on DNA extracted from the metastatic tissue. No mutations for BRAF exon 15, NRAS exon 2, KRAS exon 2, HRAS exon 2, and PIK3CA exons 9 and 20 were detected by direct sequencing. In addition, the search for BRAF V600E mutation by real-time PCR was also negative. 

In August 2011, a radioiodine treatment with thyroid hormone withdrawal (THW) was done. The patient received two treatments with 3.7 GBq 131-I each. The subsequent posttherapeutic whole-body scans showed no uptake other than basic thyroid deposits from the first treatment. The thyroglobulin (Tg) level in THW was initially at 0.4 *μ*g/l with a TSH of 75 *μ*UI/ml at the first treatment and undetectable with a TSH of 63 *μ*UI/ml during the second treatment by 131-I. There was no iodated saturation during the different treatments. The antithyroid antibodies were also normal. Since treatment, the patient is in complete clinical, biological remission (Tg < 0.1 *μ*g/l). 

## 3. Discussion

Thyroid cancer is rapidly becoming a frequent cancer with an incidence of 8.1% per year in women and 6.2% in men, mainly due to an increase in papillary types, with an epidemic of microcarcinomas [4]. According to ATA and ETA Guidelines as regards the PTMCs, treatment consists of total thyroidectomy or partial surgery [5]. While most PTMCs behave in a relatively nonaggressive fashion, some patients will have poor outcomes [6, 7]. Clinical and histological risk factors that may suggest possible malignant behavior (essentially local recurrence) have been identified in the medical literature such as age older than 45 years, size >5 mm, multifocal carcinoma, extrathyroid extension, vascular invasion, node involvement, and BRAF V600E mutations [2, 8–11]. In this case, except age there are no poor prognosis factors. 

In the medical literature, only a few cases of metastatic PTMCs have been described in the literature [12]. Mercante et al. [13] report four metastatic cases in a series of 445 PTMCs, all these metastases had extension outside the thyroid or lymph node involvement. In a series of 190 patients, Sugitani et al. [14] describe 2 metastatic patients (with no bone involvement). They both had macroscopic lymph node involvement and secondary pulmonary involvement. Finally, among 207 PTMC patients, Baudin et al. [15] describe 8 metastatic cases: 7 with a multifocal thyroid carcinoma and all with lymph node involvement on initial presentation. The location of the metastases was primarily pulmonary, then cerebral (5/8 and 2/8, respectively). Only one case of bone metastasis was reported.

Further, even when small in size, follicular thyroid carcinomas have poor prognosis and can develop bone metastases [16] probably due to the frequency of arterial embolisms. However, follicular variants of papillary carcinomas (FVPCs) appear to have a better prognosis. In a retrospective study covering 114 cases of encapsulated standard and follicular variants of PTC, Baloch et al. observed a very low mortality with only three metastatic cases and an excellent prognosis [17]. In a group of 78 patients with FVPC, Liu et al. found two distinct groups: a non-encapsulated subvariant with higher risk of lymph node involvement (11 of 17 patients) and an encapsulated form that showed no higher risk for lymph node or distant metastase [18]. Finally, in a series of 36 PTMCs, Kunavisarut et al. found a modification of the expression of the epithelial cell molecule (EpCAM) in the papillary cancers. The reduction of the extracellular domain and the increase of the nuclear and cytoplasmic domains of the EpCAM were found to correlate positively with aggressive PTMCs [19].

## 4. Conclusions

Here, we present an extremely rare case of a PTMC revealed by a single costal bone metastasis with no poor prognosis histological factors. This case highlights the need for continued characterization research in PTMCs that generally are nonaggressive and have excellent prognosis. It also demonstrates that a metastasis of adenocarcinoma with immunostaining positive for TTF1 and thyroglobulin indicates a thyroid origin.
